# Uterine and systemic inflammation influences ovarian follicular function in postpartum dairy cows

**DOI:** 10.1371/journal.pone.0177356

**Published:** 2017-05-19

**Authors:** Soon Hon Cheong, Ocilon G. Sá Filho, Victor A. Absalon-Medina, Augusto Schneider, W. R. Butler, Robert O. Gilbert

**Affiliations:** 1 Department of Clinical Sciences, College of Veterinary Medicine, Cornell University, Ithaca, New York, United States of America; 2 Department of Animal Science, Cornell University, Ithaca, New York, United States of America; University of Florida, UNITED STATES

## Abstract

The objective of this study was to determine the effects of uterine and systemic inflammatory responses to uterine bacterial contamination at calving in dairy cows on the growth and ovulatory outcomes of the first dominant follicle postpartum. Ovulatory capability of the first dominant follicle postpartum was predicted in 53 multiparous cows by using a combination of follicle growth characteristics and circulating estradiol concentrations. Endotoxin levels were assayed in follicular fluid samples that were aspirated the day after ovulatory outcome prediction. Plasma levels of haptoglobin, a proinflammatory acute phase protein, and paraoxonase, a negative acute phase protein were determined. Uterine bacteria and inflammation were evaluated in three uterine fluid samples from each cow collected on the day of calving, the day after follicle aspiration, and at 35 days postpartum. Cows that had a strong initial uterine inflammatory response (robust recruitment of polymorphonuclear leukocytes of ≥ 35% and cows with uterine pH < 8.5 on the day of calving) were more likely to have an ovulatory first dominant follicle. Follicular fluid endotoxin levels were higher in non-ovulatory cows compared with ovulatory cows. Endotoxin levels in circulation were not different between ovulatory groups but were higher prepartum than on day 7 and 14 postpartum. Systemic inflammation characterized by elevated haptoglobin concentrations was higher in non-ovulatory cows despite similar bacterial contamination and circulating endotoxin levels. Paraoxonase activity in follicular fluid was significantly associated with the paraoxonase activity in plasma, however, plasma paraoxonase concentrations were not different between non-ovulatory and ovulatory cows. Cows with a higher uterine bacterial load on the day of calving had slower ovarian follicle growth. In summary, a robust uterine inflammatory response on the day of calving was positively associated with ovarian function while elevated systemic inflammation during the early postpartum period was negatively associated with the ovulatory status of the first dominant follicle postpartum.

## Introduction

The fertility of lactating dairy cows is poor compared with that in non-lactating animals [1]. After calving, dairy cows must overcome two primary challenges to have good fertility later during breeding: namely restoring uterine health and resuming ovulatory ovarian follicular function [2,3]. A healthy uterine environment allows sperm cells to traverse the reproductive tract successfully to reach the site of fertilization and allows the embryo to develop in a favorable environment [4]. The ovary must also have appropriate follicular function to produce a developmentally competent oocyte and to create a suitable endocrine environment for breeding and pregnancy [1]. These two reproductive processes are distinct, but clearly, there are interactions between uterine health status and ovarian follicular functions. Early resumption of ovarian cyclicity postpartum results in improved reproductive performance [5,6]. Each ovulation increases in fertility [7] and cows that have an early resumption of ovulation would have more cycles before being bred for the first time and thus are more likely to become pregnant.

The reproductive tract is exposed to trauma and microbial challenges at calving and during the early postpartum period. Most cows will have bacterial contamination of the uterus [8] which is eventually cleared by the immune system in healthy cows. While virtually all cows have intrauterine inflammation in the early postpartum period, a smaller proportion of cows will have a systemic immune response characterized by elevation of acute phase proteins such as haptoglobin [9–11]. The release of acute phase proteins by the liver can be stimulated by bacterial products such as endotoxin which can also inhibit the production of paraoxonase that has anti-inflammatory functions [9,12]. All of these factors, the presence of bacteria and bacterial products in the uterus, elevated acute phase proteins and decreased anti-inflammatory proteins are associated with poor uterine health.

The ovarian ovulatory function is also affected by the same factors related to poor uterine health. Cows with more bacterial contamination in the uterus had slower follicular growth and decreased estradiol production [8,13]. Bacterial endotoxin can directly inhibit estradiol production by granulosa cells in vitro [13,14]. Impaired estradiol production is a critical difference between follicles that do not ovulate early postpartum *versus* ovulatory follicles [15–17].

Injecting cows with endotoxin causes a systemic inflammatory response characterized by fever and decreased paraoxonase in serum [18] and can also indirectly affect follicle function by depressing luteinizing hormone (LH) pulse secretion from the anterior pituitary [19]. Decreased LH pulse frequency is an important feature of non-ovulatory cows [17,20]. Cows that ovulated before 44 days postpartum tended to have lower prepartum haptoglobin levels and higher paraoxonase and albumin levels postpartum than non-ovulatory cows [9].

There is an intimate anatomic relationship between the utero-ovarian vein and the ovarian artery in cattle that allows the counter-current transfer of prostaglandins produced by the uterus to pass from the vein into the ovarian artery concentrating the prostaglandin product in the ovary [21]. This same counter-current transfer mechanism potentially exposes the ovary to higher concentrations of bacterial and other inflammatory products from the uterus during the early postpartum period. Endotoxin has been shown to be present in much higher concentrations in follicular fluid compared with the general circulation [13,22]. Therefore, inflammation of the reproductive tract may have a strong direct effect on the ovarian follicular environment.

Evaluation of uterine contents may also be helpful in understanding the relationship between uterine health and ovarian function. Most cows have some bacteria in the uterus early postpartum, but the type of bacteria present may be important. Some bacterial species have been negatively associated with fertility such as *Escherichia coli*, *Trueperella pyogenes*, *Fusobacterium necrophorum*, and *Prevotella melaninogenica* while the presence of alpha-hemolytic streoptococcus early postpartum appears beneficial [8,23,24]. Intrauterine inflammation is usually diagnosed by cytological evaluation of cells in uterine samples [25–27]. A higher proportion of polymorphonuclear leukocytes (PMN) in uterine samples associated with robust initial recruitment of PMN hastens resolution of uterine inflammation postpartum [23]. Cows with intrauterine inflammation persisting beyond 35 days after calving are considered to have endometritis [28]. Cows with endometritis are more likely to be anovulatory, and there is an additive negative effect of anovulation and endometritis on subsequent fertility [28,29]. The timing of exposure is an important consideration for understanding the relationship between uterine health and ovarian follicular function. As uterine challenges occur at parturition and the first follicular wave begins approximately three days postpartum, uterine health may have a major role in determining follicle function, in particular, the ovulatory capability of the first dominant follicle postpartum. Cows that ovulate when the uterus has heavy bacterial contamination may also develop pyometra, a disease characterized by the accumulation of pus in the uterus [30]. Administration of exogenous gonadotropin-releasing hormone early postpartum to induce ovulation can predispose cows to develop pyometra [31] but induction of ovulation by treatment with gonadotropin-releasing hormone early postpartum did not affect fertility in artificially inseminated cows [32].

The objective of this study was to determine the effects of uterine and systemic inflammatory responses to uterine bacterial contamination at calving in dairy cows on the growth and ovulatory outcomes of the first dominant follicle postpartum.

## Materials and methods

### Animals

Holstein cows from the Cornell Teaching and Research Dairy Unit were used in this study. A total of 53 multiparous cows were enrolled for the study 28 days before the projected calving date and studied until 35 days after calving. Cows were housed in tie-stalls with free access to water and individually fed total-mixed rations. Rectal temperature was measured daily starting from 10 days before the due date and fever was defined as a rectal temperature of ≥ 39.5°C. Acute puerperal metritis was defined as fever with reddish-brown foul-smelling vaginal discharge. Cows were considered to have retained fetal membranes if the fetal membranes were not passed within the first 24 hours postpartum. All procedures were approved by the Cornell University Institutional Animal Care and Use Committee.

### Follicle function evaluation

The two follicle functions that were evaluated as outcomes of interest were the ability of the first dominant follicle postpartum to ovulate and the rate of follicle growth. The outcome of the first dominant follicle postpartum was determined and described as being ovulatory or non-ovulatory based on a combination of follicle growth characteristics and estradiol production [17]. Ovarian structures were measured daily by transrectal ultrasound using a 7.5 MHz linear probe (Aloka Inc. Wallingford, CT) starting 7 days postpartum and followed until follicles were aspirated (average on day 14 postpartum). Follicle sizes were measured using internal calipers, and the location on the ovary was mapped for all follicles ≥ 5 mm. The first follicle to reach 10 mm in diameter was considered the first dominant follicle postpartum. Circulating estradiol concentrations were measured daily. Follicles in cows were considered ovulatory (**OV**), if circulating estradiol was ≥ 2 pg/ml in the presence of a follicle > 10mm in diameter. These criteria of ≥ 2 pg/ml of circulating estradiol and follicle diameter of > 10 mm were selected for the strong correlation with eventual ovulation when evaluating the follicular growth patterns of the first-dominant follicle postpartum and estradiol levels in cows from previous studies [15,33]. The follicle was considered non-ovulatory (**NOV**), if the first-dominant follicle postpartum failed to grow (i.e., same or decreased internal diameter of the largest follicle on consecutive days measured by ultrasound) or if circulating estradiol did not reach ≥ 2 pg/ml by the day after the follicle reached a diameter of 15 mm. Follicular fluid from the first dominant follicle postpartum was collected by ultrasound guided fine-needle aspiration the day after reaching one of the following criteria: 1) circulating estradiol concentrations reached ≥ 2 pg/ml, 2) follicle failed to grow on daily ultrasound examination after reaching a diameter of > 10 mm, or 3) follicle reached a diameter of > 15 mm. A subset of nine cows (4 OV and 5 NOV) were not aspirated to validate the prediction of ovulatory fate, and the follicle fate was accurately predicted in all 9 of these cows as previously described [17].

### Hormone and systemic inflammatory markers

Circulating estradiol concentrations were determined daily starting day 7 postpartum using RIA (Serono-Maia, Cortland Manor, NY) as previously described [17]. Endotoxin concentrations in follicular fluid were determined using a commercial kit (Endpoint Chromogenic LAL Assay, Lonza Walkersville Inc, Walkersville, MD) as previously described [13,34]. Follicular fluid samples were diluted 1:50 and heat inactivated at 75°C for 30 minutes. Each sample was serially diluted until the values were within the standard curve range. Each dilution was tested by spiking the samples with a known quantity of endotoxin. Recovery of >80% was considered to have no inhibition of the reaction. Plasma samples on day 7 prepartum, and on days 1, 7 and 14 postpartum were evaluated for endotoxin levels. Samples with endotoxin levels below the detection limit were considered for statistical evaluation to be at the lowest detection limit which was 0.1 EU/mL.

To monitor systemic inflammation, plasma concentrations of the acute phase protein haptoglobin were determined in all cows, and the anti-inflammatory protein paraoxonase was determined in a subset of 20 cows (10 OV and 10 NOV cows). The first 10 cows that fit into the respective grouping criteria for OV and NOV were selected. Plasma samples on day -21, -16, -9, -3, 0, 3, 9 and 15 from calving were analyzed for each cow. These samples were selected to represent the majority of the transition period from weeks 3, 2, and 1 prepartum, and weeks 1 and 2 postpartum known to be a critical time for dairy cows to adjust from a late pregnancy state to lactation, with higher temporal resolution around the calving date. Plasma haptoglobin concentration was determined in plasma samples using a guaiacol test as previously described [35]. Paraoxonase levels in plasma and follicular fluid were determined using a commercial kit (OXItek, ZeptoMetrix Corp. Buffalo, NY) [11]. Intra and inter-assay coefficients were 8.8% and 16%; and 9.1% and 5%, respectively, for haptoglobin and paraoxonase assays.

### Uterine inflammation and bacteriology

Uterine samples were collected three times for each cow using low-volume uterine lavage [26] previously shown to have minimal effects on future fertility [36]. The first sample was collected on the day of calving, the second sample was collected the day after follicle aspiration ± 1 day, and the third sample was collected at 35 ± 1 days after calving. The perineum was cleansed and a sterile pipette introduced through the cervix into the uterus. Sterile saline (20 ml) was infused into the uterus, and the uterus was massaged per rectum before recovery.

Uterine inflammatory status was determined by evaluating the proportion of inflammatory cells in uterine samples by cytology and by using a reagent strip test. Uterine cytology was performed on the uterine samples as previously described [37]. Cytology slides were prepared using a cytocentrifuge and stained with a commercial Romanowsky stain (Camco Stain Pak, Cambridge Diagnostic Products Inc., Fort Lauderdale, FL). Two hundred cells were counted excluding erythrocytes, and the proportions of polymorphonuclear (PMN) cells (which were mostly neutrophils), uterine epithelial cells, lymphocytes, and macrophages were determined. Inflammatory markers in uterine lavage samples known to be associated with reproductive performance were tested using a urinary reagent strip Multistix 10 SG (Bayer Corp., Elkhart, IN) immediately after collection by placing a drop of recovered fluid directly on each reagent strip [37]. The reagent tests evaluated were leukocyte esterase test which detects a leukocyte-specific enzyme, a protein which should be elevated in inflammatory exudate, and pH which is elevated in inflamed secretions of cattle [37–39].

Bacterial content of the uterine lavage samples was determined. A sterile swab was soaked in the uterine sample for approximately 5 minutes then put into transport media (BBLTM Port-A-CulTM Tube, Becton Dickinson and Co., Sparks MD) and submitted to the Animal Health Diagnostic Center at Cornell University for aerobic and anaerobic bacteria, mycoplasma and ureaplasma culture. Anaerobic bacteria culture was not performed when samples could not be submitted to the laboratory promptly *i*.*e*. during holidays and weekends.

Aerobic cultures were performed by first inoculating samples onto Columbia agar with 5% sheep blood, chocolate agar, eosin methylene blue agar, and colistin/nalidixic acid agar plates. After 24 to 48 hours of growth, bacterial colonies of varying morphologies were tested using manual biochemical tests. Complete identification was done using the Sensititre automated bacterial identification system (TREK Diagnostic Systems Inc., Cleveland, OH) used according to the CLSI guidelines. Anaerobic cultures were performed by inoculating samples onto Brucella agar, phenyl ethyl alcohol agar, Laked Blood with Kanamycin and Vancomycin agar, and Bacteroides Bile Esculin Agar plates. Bacterial isolates were confirmed as strict anaerobes by failure to grow in the presence of oxygen and identified using a combination of staining and resistance characteristics. Semi-quantitative bacterial concentration data was collected for each bacterial isolate and the categories recorded were: Few, Moderate, and Many. *Mycoplasma* spp. and *Ureaplasma* spp. were cultured from samples by inoculation onto agar media specific for the growth of these organisms and identified visually using a dissecting microscope. For ureaplasma, samples were also inoculated into ureaplasma broth and were subcultured if the appropriate color change was observed. All *Escherichia coli* isolates were saved for phylogenetic typing.

Phylogenetic group (A, B1, B2 or D) were determined for the *E*. *coli* isolates using PCR as previously described [40]. Isolated bacteria were classified as known uterine pathogens: *Trueperella pyogenes*, *Escherichia coli*, *Fusobacterium necrophorum*, *Fusobacterium nucleatum*; potential uterine pathogens: *Histophilus somni*, *Mannheimia haemolytica*, *Pasteurella multocida*, *Peptostreptococcus spp*., *Streptococcus uberis*, *Staphylococcus aureus*; and bacteria not recognized as uterine pathogens [8]. *Mycoplasma* spp. and *Ureaplasma* spp. were tested individually.

Total bacterial load score for each uterine sample was determined by adding one point for each bacterial isolate with the semi-quantitative description of “Few” colonies, two points for each bacterial isolate with a “Moderate” colony density, and three points for each bacterial isolate with “Many” colonies in aerobic, anaerobic, mycoplasma and ureaplasma culture. Therefore, a cow that had “Moderate” growth of *Escherichia coli* (contributing 2 points) and “Few” α-hemolytic Streptococcus (contributing one point) on aerobic culture, and no growth on anaerobic culture, mycoplasma culture, and ureaplasma culture (contributing 0 points) would have a bacterial load score of “3”. For the bacterial load on the day of calving, the aerobic bacterial load score was used instead of total bacterial load score as 28% of the cows calved on a weekend or holiday and did not have an anaerobic culture performed.

### Statistical analysis

The association between uterine inflammation and ovulatory function was determined using the LOGISTIC procedure of SAS ver. 9.4 (SAS Institute Inc., Cary, NC). Uterine sampling results were tested individually for association with ovulation. Continuous outcomes were dichotomized if the outcome was associated with ovulatory outcome at *P* < 0.20. A receiver operator characteristics curve (**ROC**) was used to determine the optimal cutoff point in JMP Pro version 12 (SAS Institute Inc., Cary, NC). Covariates tested were parity, calving-ease score, calf sex, acute puerperal metritis, and retained fetal membranes. The final model was built using a backward stepwise method and variables retained if *P* < 0.05.

Repeated measures analysis was used when evaluating dependent variables that were measured at several time points for each cow such as follicle size (to evaluate follicle growth), plasma haptoglobin, paraoxonase, and endotoxin. The MIXED procedure of SAS ver. 9.4 with first order auto-regressive covariance structure was used. Two-way interactions were tested for all models, and when significant, a posthoc multiple comparison test using Bonferroni correction was performed. Bacterial load score effects on follicular size were tested initially as a continuous variable and were dichotomized if the bacterial load score was associated with follicle size at *P* < 0.20. The threshold for bacterial load score was determined by the model with the lowest Akaike inclusion criterion.

Bacterial load association with follicle outcome was tested for individual uterine sampling time point using PROC LOGISTIC of SAS ver. 9.4. Bacterial load score was tested as a continuous variable and if dichotomized at the optimal threshold using ROC in JMP Pro version 12. Bacterial isolates were also tested grouped by the presence of at least one known pathogenic bacteria species in any of the three uterine sample time points and tested again including potential pathogenic bacteria isolates using PROC LOGISTIC of SAS ver. 9.4. Distribution of continuous variables was tested and transformed as necessary to fit model assumption. Variables were considered significant if *P* < 0.05 and considered a tendency when *P* < 0.10.

## Results

### Uterine inflammation and ovulation

The proportion of PMN in the first uterine sample, as a continuous variable, and the pH of the first uterine sample, also as a continuous variable, tended to be associated with follicle outcome. A ROC analysis was performed to identify the optimal cutoff point which was found to be at 35% PMN and pH 8.5. Cows that had ≥ 35% PMN in the first uterine sample were more likely to be OV (OR = 5.04, 95% C.I. 1.20–21.06; *P* = 0.016) and cows that had the first uterine sample with pH < 8.5 were also more likely to be OV (OR = 5.13, 95% C.I. 1.53–17.28; *P* = 0.008). All other reagent strips and cytological outcomes in any of the three uterine samples collected from each cow were not associated with follicle status or follicle size. There was no association between the other covariates: parity, calving-ease score, calf-weight or their interactions with follicle outcome or follicle size. There was a tendency for cows that had bull calves to be NOV (OR = 2.80, 95% C.I. 0.89–8.86; *P* = 0.080). Retained fetal membranes were not associated with follicle capability for ovulation, but cows that developed acute puerperal metritis tended to be NOV (OR = 3.60, 95% C.I. 0.94–13.78; *P* = 0.062). A multivariable model was tested, but did not retain any variables with *P* < 0.05.

### Systemic inflammation and ovulation

**Fig 1:**
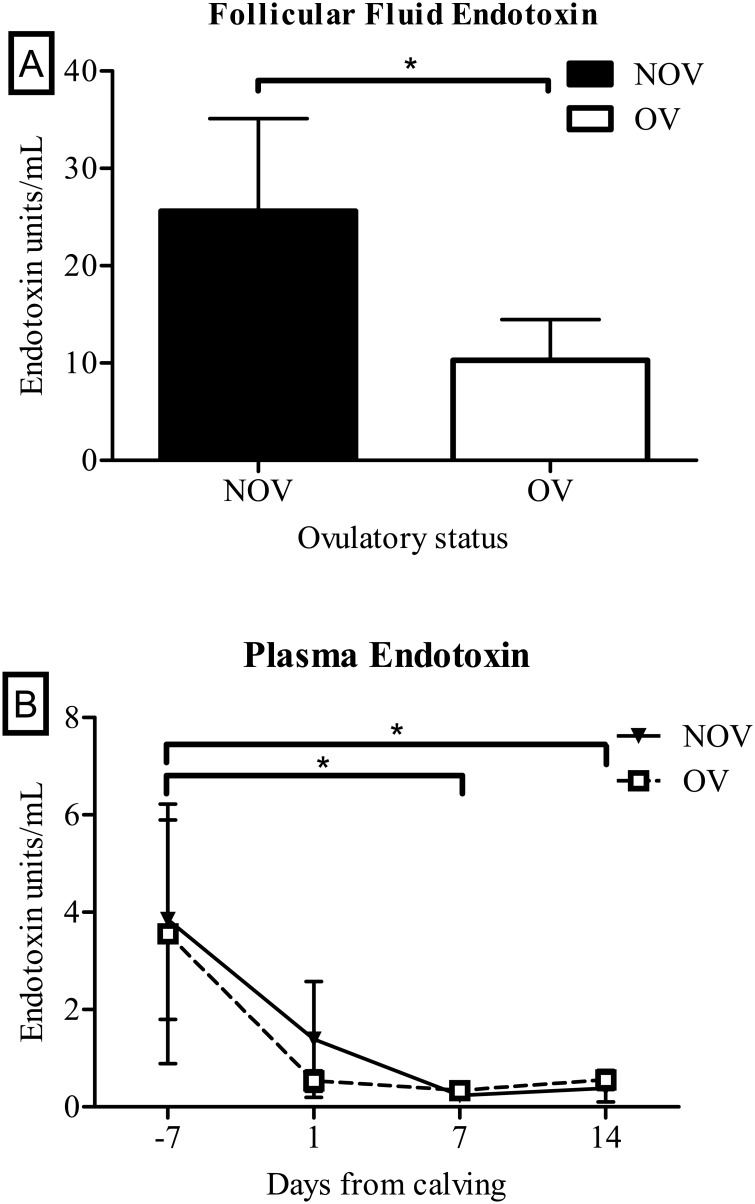
Endotoxin levels in follicular fluid and in circulation. A) Endotoxin levels in follicular fluid were higher in NOV cows compared with OV cows (n = 43, * = *P* < 0.05). B) Plasma endotoxin levels were not different between NOV and OV cows but were higher (*, *P* < 0.05) on day 7 prepartum compared with day 7 and 14 postpartum.

Follicular fluid endotoxin levels were below the assay detection limit in 26 of the 43 samples tested and were assigned the value of minimum detection limit of the assay which was 0.1 EU/mL. Cows with measurable endotoxin levels in follicular fluid were more likely to be non-ovulatory (OR for NOV = 4.99 per EU/ml endotoxin, 95% C.I. 2.87–13.96; *P* = 0.038; Fig 1A). There was no difference in plasma endotoxin levels between OV and NOV cows, but overall plasma endotoxin levels were higher (*P* < 0.05) on day -7 (seven days before calving) compared with the endotoxin levels at day 7 and 14 after calving (Fig 1B).

**Fig 2:**
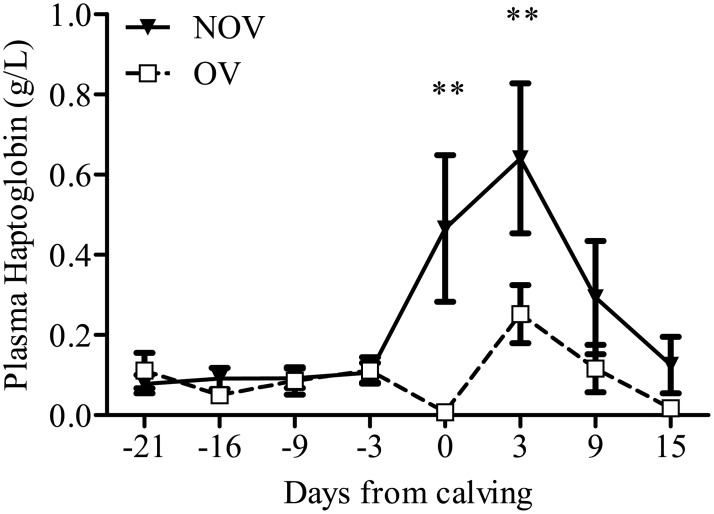
Plasma haptoglobin for ovulatory (OV) and non-ovulatory (NOV) cows. NOV cows have significantly higher levels of haptoglobin compared with OV cows.

Even though there were no differences in circulating endotoxin levels between NOV and OV cows, there was a difference in the acute phase protein haptoglobin concentrations between NOV and OV cows where NOV cows had 0.22 ± 0.09 g/L higher haptoglobin compared with OV cows from the repeated measures analysis (*P* = 0.026). There were no significant interactions (*P* = 0.448) between days from calving and follicle outcome on plasma haptoglobin (Fig 2).

**Fig 3:**
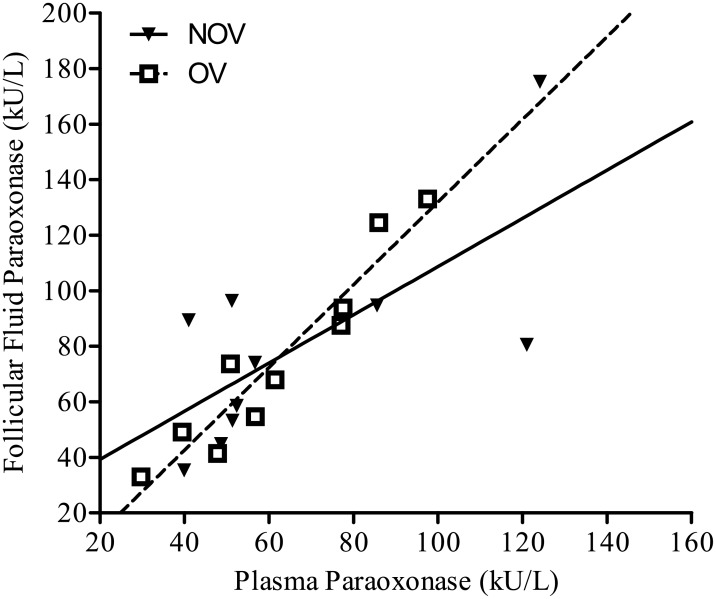
Scatterplot for follicular fluid and plasma paraoxonase. The association between the follicular fluid and plasma paraoxonase concentrations was linear and significant (*P* < 0.001). No differences were detected between OV and NOV groups.

Paraoxonase activity in follicular fluid was significantly correlated (*P* < 0.001) with the paraoxonase activity in plasma (Fig 3). There were no significant interactions between days from calving and follicle outcome with plasma paraoxonase activity. Plasma paraoxonase was not different (*P* = 0.50) between NOV and OV cows.

### Bacteriology and ovarian function

The proportion of cows that had at least one bacterial colony isolated from uterine cultures decreased from 50/53 to 41/53 to 27/53 on the day of calving, the day after aspiration and at 35 days postpartum, respectively. The list of bacteria cultured from the uterine samples and the number of samples cultured positive for each bacterial species are summarized in Tables 1–3. There were no significant associations between the presence of any individual bacterial species in any of the three uterine sampling time points on follicle outcome. Grouping bacterial species by pathogenicity, 73.1% of cows had pathogenic bacteria in at least one of the uterine cultures, and when potential pathogens were included, 80.8% of cows had known or potential pathogens isolated from uterine samples. The association between follicle outcome and the presence of a known bacterial pathogen (OR = 1.83, 95% C.I. 0.53–6.33; *P* = 0.34) or combined known and potential bacterial pathogen (OR = 0.50, 95% C.I. 0.12–2.04; *P* = 0.33) were not significant. All cows that had mycoplasma or ureaplasma isolated from uterine samples also had a known pathogenic bacteria isolated in at least one of the uterine samples.

**Table 1:** Number of samples that cultured positive for each type of aerobic bacterial species isolated from uterine samples on the day of calving (sample 1), the day of follicular fluid aspiration ± 1 (sample 2), and on 35 ± 1 days postpartum (sample 3).

Phylum	Genus	Sub-classification	Species	Sample 1 (n = 53)	Sample 2 (n = 52)	Sample 3 (n = 52)
Actinobacteria	Trueperella		Trueperella pyogenes	4	16	3
	Corynebacteria		spp.	1	2	4
Firmicutes	Bacillus		spp.	1	0	2
	Staphylococcus		spp.	0	2	1
		Coagulase-negative	Coagulase-negative Staphylococcus	1	0	1
			Staphylococcus haemolyticus	0	1	0
		Coagulase positive	Staphylococcus intermedius	1	0	0
	Enterococcus		Enterococcus faecium	4	0	0
	Streptococcus		spp.	3	0	0
		α-hemolytic	α-hemolytic Streptococcus	28	4	10
		β-hemolytic	Streptococcus dysgalactiae	0	2	0
			Streptococcus bovis	1	0	0
		Non-hemolytic	Streptococcus uberis	4	4	3
Proteobacteria	Escherichia	Not pathotyped	Escherichia coli	1	2	1
		Pathotype A	Escherichia coli	1	0	0
		Pathotype B1	Escherichia coli	22	7	1
		Pathotype B2	Escherichia coli	2	1	0
		Pathotype D	Escherichia coli	2	2	1
			Escherichia fergusonii	2	0	0
	Proteus		Proteus mirabilis	0	2	0
	Serratia		Serratia plymuthica	0	0	0
	Actinobacillus		spp.	1	0	0
	Histophilus		Histophilus somni	0	0	1
	Mannheimia		Mannheimia haemolytica	2	0	0
	Pasteurella		spp.	1	1	1
	Moraxella		Moraxella osloensis	0	1	1
No growth of aerobic bacteria				4 (7.5%)	13 (25%)	29 (55.8%)

**Table 2:** Number of samples that cultured positive for each type of anaerobic bacterial species isolated from uterine samples on the day of calving (sample 1), the day of follicle fluid aspiration ± 1 (sample 2), and on 35 ± 1 days postpartum (sample 3).

Phylum	Genus	Species	Sample 1 (n = 38)	Sample 2 (n = 52)	Sample 3 (n = 52)
Actinobacteria	Actinomyces	spp.	1	4	3
		Actinomyces viscosus	1	0	0
	Eggerthella	Eggerthella lenta	1	0	0
	Propionibacterium	Propionibacterium acnes	0	1	1
Firmicutes	Clostridium	spp.	3	0	0
		Clostridium perfringens	8	2	0
	Peptostreptococcus	spp.	4	1	4
		Peptostreptococcus asaccharolyticus	2	2	1
		Peptostreptococcus anaerobius	0	0	1
Bacteroidetes	Bacteroides	Bacteroides fragilis	0	1	0
		Bacteroides ovatus	0	1	0
		Bacteroides vulgatus	0	1	0
	Porphyromonas	spp.	0	0	1
	Prevotella	spp.	1	1	0
Fusobacteria	Fusobacteria	spp.	0	1	0
		Fusobacterium necrophorum	0	2	2
		Fusobacterium nucleatum	1	0	0
		Fusobacterium varium	0	2	1
No growth of anaerobic bacteria			23 (60.5%)	37 (71.2%)	39 (75%)

**Table 3:** Number of uterine samples that cultured positive for mycoplasma and ureaplasma collected on the day of calving (sample 1), the day of follicle aspiration ± 1 (sample 2), and at 35 ± 1 days postpartum (sample 3).

Phylum	Genus	Species	Sample 1 (n = 53)	Sample 2 (n = 52)	Sample 3 (n = 52)
Tenericutes	Mycoplasma	spp.	3	6	2
	Ureaplasma	spp.	2	8	1

### Bacterial effects on follicle size

Bacterial load score in the first uterine sample was significantly associated with follicle size (*P* = 0.02). Threshold for bacterial load score in the first uterine sample associated with follicle size was ≥ 4 based on the lowest Akaike inclusion criterion and cows that had bacterial load score ≥ 4 had smaller follicles (0.40 mm, 95% C.I. 0.74–0.06; *P* = 0.02). Bacterial load score in the second and third uterine samples were not associated with follicle size (*P* > 0.10). Uterine bacterial load score was not significantly associated with ovulatory status in any of the uterine samples and the presence of at least one known pathogenic bacteria in any of the three uterine samples was also not significantly associated with ovulatory status.

The presence of *E*. *coli* on the day after calving was significantly associated with lower proportion of PMN in the same uterine sample (61.2% ± 4.9 versus 48.8% ± 4.8; *P* = 0.012). The association between the presence of *E*. *coli* and PMN was not significant in the second and third uterine samples.

## Discussion

In the present study, cows with robust initial recruitment of PMN into the uterus and low uterine fluid pH on the day of calving were more likely to have ovulatory first dominant follicles. Cows that have stronger initial recruitment of inflammatory cells into the uterus have been shown to have a faster resolution of uterine inflammation postpartum [23]. On the other hand, elevated uterine fluid pH was associated with prolonged uterine inflammation when sampled between 40–60 days postpartum [37]. It is possible that uterine inflammation characterized by infiltration of inflammatory cells into the uterine lumen on the day of calving elicits a different effect on uterine pH compared with uterine inflammation later postpartum. Uterine inflammation on the day of calving was associated with ovulation of the first dominant follicle postpartum, but uterine PMN, leukocyte esterase, pH, and protein were not different between OV and NOV cows in the subsequent uterine sampling periods. High proportion of PMN in uterine samples in the first 3 weeks postpartum is poorly correlated with future fertility, but elevated proportions of PMN after day 35 postpartum is associated with reduced fertility [28,41,42] and delayed resumption of ovarian cyclicity [6,29,41,43].

Endotoxin levels were higher in follicular fluid of NOV cows compared with OV cows but were similar in plasma between NOV and OV cows at all time points. The source of endotoxin postpartum may be from the uterus which is contaminated by bacteria in the majority of cows. Endotoxin can be absorbed from the uterine lumen into circulation [19] then pass through follicle walls into the follicular fluid. Cows with uterine infections have been reported to have high endotoxin levels [13]. The effects of endotoxin on steroidogenesis include the impairment of aromatization of androgens to estradiol without affecting androgen production [13,14]. In NOV cows, the follicular fluid levels of estradiol were low, but the levels of androstenedione were also low [17]. There may be inhibition of aromatase in NOV cows. However, the inhibitory effects of endotoxin on follicle estradiol production may be primarily through impaired gonadotropin secretion *i*.*e*.,. NOV cows had lower LH pulse frequency compared with OV cows [17]. Endotoxin administration has been shown to impair both GnRH and LH release in sheep [44]. Perhaps the most surprising finding regarding endotoxin was the higher circulating endotoxin levels on day 7 before calving compared to the endotoxin level in circulation on day 14 postpartum. The source of the higher endotoxin in circulation during late pregnancy is unknown but could be the gastrointestinal tract or the mammary gland [45,46]. The gastrointestinal tract is a likely source as it is a large organ filled with bacteria that can enter the bloodstream. The mammary gland is susceptible to new bacterial infections during the dry period [47], but infected cows do not develop clinical signs of mastitis until after calving [46]. The immune response of cows during pregnancy is biased towards the T-helper type 2 environment and responds to the infection by secreting the anti-inflammatory cytokine interleukin-10 [45]. High levels of interleukin-10 prevent the release of pro-inflammatory cytokines tumor necrosis factor α and interleukin-1β which allow the bacterial infection in the mammary glands to remain dormant [45]. At calving, the immune environment shifts to a T-helper type 1 response and the mammary macrophages secretes the pro-inflammatory cytokines in response to the latent bacterial infection that results in the liver producing acute phase proteins such as haptoglobin.

The bacterial contamination of the uterus postpartum and circulating endotoxin levels was similar between NOV and OV cows, but there were differences in the systemic inflammatory response during the early postpartum period. Plasma haptoglobin levels were significantly higher in NOV than OV cows on the day of calving and at 3 days postpartum. This temporal pattern observed for haptoglobin levels is similar to that in cows with uterine disease [10]. High haptoglobin levels early postpartum have also been reported in cows with high uterine bacterial load [34]; however, the association between haptoglobin and uterine bacterial load at any of the three sampling times was not significant in the current study (*P* > 0.20). Elevated haptoglobin levels is an indication of a heightened systemic inflammatory state in NOV cows compared with OV cows, but cytology of the uterine contents on the day of calving to evaluate inflammatory status indicated that the proportion of PMN was lower in NOV cows. Therefore, systemic inflammation is detrimental for early resumption of ovarian cyclicity, but a more robust local inflammatory response (increased PMN recruitment) in the uterus appears to be beneficial [41,43]. This is in agreement with studies using a cow model of fertility wherein low fertility cows express delayed first postpartum ovulation [48] and are less capable of regaining control of inflammation following the onset of lactation compared to high fertility cows [49].

In the present study, there was a direct linear relationship between follicular fluid and plasma paraoxonase levels in agreement with our previous report [50]. The similar levels of paraoxonase in follicular fluid and plasma suggest passive transfer of paraoxonase from blood into the follicular fluid, unlike endotoxin which is present in much higher concentrations in the follicular fluid than in blood plasma. Paraoxonase production is negatively correlated with haptoglobin and other positive acute phase proteins [11,12]. However, no difference was observed between OV and NOV groups in the present study.

The presence of *E*. *coli* in the uterus is associated with reduced proportion of PMN in uterine cytology on the day of calving. This observation is consistent with clinical evaluation of mares with *E*. *coli* isolated from uterine samples, which have fewer PMN present in uterine cytology compared with mares that have *Streptococcus equi subspecies zooepidemicus* [51]. Additionally, mares with positive uterine culture had lower pregnancy rates compared with controls even when the uterine cytology did not show elevated proportion of PMN [51].

## Conclusions

We conclude that robust initial intrauterine inflammation on the day of calving is beneficial for ovarian ovulatory status while the heightened systemic inflammatory response to uterine bacterial contamination at calving is detrimental for ovulatory status. Surprisingly, some cows had elevated circulatory endotoxin during late pregnancy at levels greater than the postpartum period when the uterus is known to be contaminated. All cows have higher levels of endotoxin in follicular fluid compared with circulatory levels; however, NOV cows had significantly higher follicular fluid endotoxin concentrations compared with OV cows despite having similar circulating endotoxin levels which may be a difference in the ability of endotoxin to enter the follicular fluid. Therefore, follicular fluid endotoxin concentration may be causative for NOV cows.

## Supporting information

S1 Fig Plasma paraoxonase for ovulatory (OV) and non-ovulatory (NOV) cows. There was no significant difference in plasma paraoxonase levels between OV and NOV cows and no significant interaction between group and time.
(EPS)

S1 Table Table of logistic regression results for the association between covariates with follicular fate. (PDF)
