# Supplementary material for: Uterine and systemic inflammation influences ovarian follicular function in postpartum dairy cows
Source: PLoS One. 2017 May 19;12(5):e0177356. doi: 10.1371/journal.pone.0177356 (PMC5438135; doi:10.1371/journal.pone.0177356)
Supplement: S1 Table — (PDF) [file pone.0177356.s002.pdf]

	Level	Ov (n = 28)	NOV (n= 25)	*P*
Parity				0.61
	2	14	15	
	3	8	7	
	4	6	3	
Calving Ease Score				0.70
	No assistance	20	15	
	Slight traction	7	7	
	Moderate traction	1	3	
Calf Sex				0.08
	Female	15	8	
	Male	13	17	
Calf weight (Kg)				0.30
	Average	47.04	47.12	
	SEM	1.18	1.07	
Retained Fetal Membrane				0.77
	Yes	3	2	
	No	25	23	
Metritis				0.062

	Yes	4	9	
	No	24	16	
First Uterine Lavage				
PMN ≥ 35%				0.038
	Yes	16	22	
	No	11	3	
pH < 8.5				0.008
	Yes	21	10	
	No	6	15	
Leukocyte esterase ≥ 3				0.25
	High	26	22	
	Low	1	3	
Protein ≥ 5				0.59
	High	25	24	
	Low	2	1	
